# Single-Cell Measurements of IgE-Mediated FcεRI Signaling Using an Integrated Microfluidic Platform

**DOI:** 10.1371/journal.pone.0060159

**Published:** 2013-03-27

**Authors:** Yanli Liu, Dipak Barua, Peng Liu, Bridget S. Wilson, Janet M. Oliver, William S. Hlavacek, Anup K. Singh

**Affiliations:** 1 Biotechnology and Bioengineering Department, Sandia National Laboratories, Livermore, California, United States of America; 2 Theoretical Biology and Biophysics Group, Theoretical Division and Center for Nonlinear Studies, Los Alamos National Laboratory, Los Alamos, New Mexico, United States of America; 3 Department of Pathology and Cancer Center, University of New Mexico, Albuquerque, New Mexico, United States of America; University of Iowa, United States of America

## Abstract

Heterogeneity in responses of cells to a stimulus, such as a pathogen or allergen, can potentially play an important role in deciding the fate of the responding cell population and the overall systemic response. Measuring heterogeneous responses requires tools capable of interrogating individual cells. Cell signaling studies commonly do not have single-cell resolution because of the limitations of techniques used such as Westerns, ELISAs, mass spectrometry, and DNA microarrays. Microfluidics devices are increasingly being used to overcome these limitations. Here, we report on a microfluidic platform for cell signaling analysis that combines two orthogonal single-cell measurement technologies: on-chip flow cytometry and optical imaging. The device seamlessly integrates cell culture, stimulation, and preparation with downstream measurements permitting hands-free, automated analysis to minimize experimental variability. The platform was used to interrogate IgE receptor (FcεRI) signaling, which is responsible for triggering allergic reactions, in RBL-2H3 cells. Following on-chip crosslinking of IgE-FcεRI complexes by multivalent antigen, we monitored signaling events including protein phosphorylation, calcium mobilization and the release of inflammatory mediators. The results demonstrate the ability of our platform to produce quantitative measurements on a cell-by-cell basis from just a few hundred cells. Model-based analysis of the Syk phosphorylation data suggests that heterogeneity in Syk phosphorylation can be attributed to protein copy number variations, with the level of Syk phosphorylation being particularly sensitive to the copy number of Lyn.

## Introduction

Heterogeneity in cellular regulatory system behavior is a feature of cell populations, which may be important for adaptive population-level responses to environmental perturbations. Heterogeneity in gene expression has been extensively studied. However, heterogeneity has also been observed in systems dominated by proteins and protein interactions [1]. One striking example is nuclear factor (NF)-κB trafficking: cells challenged with tumor necrosis factor α (TNFα) display quantitative differences from cell-to-cell in NF-κB nuclear localization [2], [3], [4]. Traditional means of biochemical analysis such as Western blot and enzyme-linked immunosorbent assay (ELISA) require large numbers of cells and provide only population-averaged data, which may not reflect single-cell behavior. For example, tumor suppressor protein p53 activity exhibits damped oscillations at the population level in response to DNA damage. However, single-cell experiments show pulsed responses [5].

Heterogeneity can result from intrinsic and/or extrinsic noise [6]. Intrinsic sources of noise, in the case of a genetic regulatory network, include variations in gene expression arising from fluctuations in small population sizes of active transcription factors. Extrinsic sources of noise may include variations in the cell microenvironment within a tissue. In biological experimentation *ex vivo*, heterogeneity can arise from variations in experimental conditions such as concentration or temperature gradients. To accurately measure biological heterogeneity, we would like to eliminate or minimize experimental heterogeneity. Unfortunately, precision is difficult to achieve in large-scale (bulk) systems, where cells can experience significant mass transfer limitations [7].

Microfluidic chips, on the other hand, have characteristic dimensions at the same scale as the dimension of a cell. This permits minimization of mass transfer limitations and also allows very rapid exchange of buffers and reagents thereby minimizing gradients of stimulants experienced by cells. Consequently, many microfluidic devices have been described for single-cell analysis and have allowed researchers to investigate heterogeneity in populations of cells [8], [9], [10], [11], [12], [13]. For example, Levchenko et al. introduced a microfabricated elastomer chip wherein chemostatic conditions are well maintained [11]. Covert et al. [9], [14] demonstrated the use of a versatile microfluidic cell culture system to study how single cells respond to TNFα. They found that the process of cell activation is digital at the single-cell level but graded at the population level. Most of these devices allow imaging of single cells but not quantitative biochemical measurements integrated with sample preparation. Our group has recently reported the development of microfluidic flow cytometry for identifying bacteria and measuring the activities of protein kinases, including ERK1/2 and p38 [8], [15] and for sorting macrophages infected with *Francisella tularensis* [16]. In more recent work, we integrated cell culture, sample preparation, imaging and flow cytometry into one platform to study responses of macrophages to lipopolysaccharide [17]. Building upon these advances, we here describe a method to interrogate the FcεRI pathway in mast cells with single-cell resolution using an integrated microfluidic platform. The platform combines two single-cell measurement technologies: on-chip flow cytometry and optical imaging. It enables analysis of cell signaling at single-cell resolution. The platform also integrates sample preparation with downstream analysis, minimizing errors associated with repeated transfer and buffer exchanges.

We used our microfluidic platform to monitor IgE receptor (FcεRI) signaling in the rat basophilic leukemia cell line 2H3 (RBL-2H3). Mast cells and basophils play an important role in inflammation and allergy [18], [19], [20]. Yet, little is known about cell-to-cell variability in signaling in these cells. FcεRI signaling is initiated upon aggregation of FcεRI by an extracellular crosslinking reagent. Aggregation activates membrane-associated Src-family protein tyrosine kinases such as Lyn. Lyn phosphorylates the β and γ subunit immunoreceptor tyrosine-based activation motifs (ITAMs), which provide sites for the binding and activation of Syk, a cytosolic protein tyrosine kinase. Downstream of Syk, multiple signaling molecules are phosphorylated and recruited to participate in signaling [21]. The immediate (within minutes) consequences of signaling include the mobilization of Ca^2+^ from intracellular stores and the release of histamine and other inflammatory mediators through degranulation. On a slower time scale (hours), signaling leads to the *de novo* synthesis of cytokines and chemokines [22]. We examined signaling events spanning Syk phosphorylation, Ca^2+^ mobilization, and TNFα production at the level of single cells. Additionally, we monitored degranulation at the population level to ensure that the secretory responses obtained under our conditions are comparable to those seen under standard conditions, and we used computational modeling to investigate the origin of the heterogeneity in Syk phosphorylation that we observed.

## Results and Discussion

**Figure 1:**
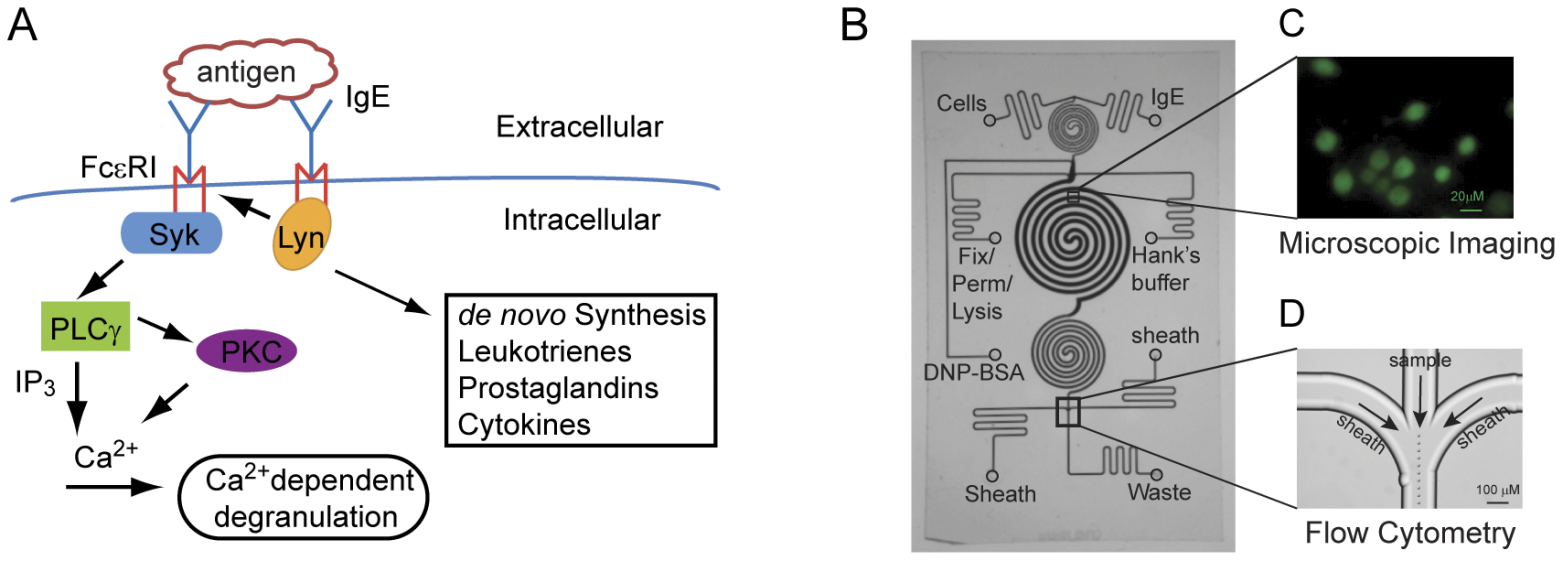
A microfluidic chip for measuring IgE-mediated FcεRI signaling events. A) General scheme of FcεRI signaling. Crosslinking FcεRI, the high-affinity IgE receptor, through IgE-FcεRI interaction with mulitvalent antigen activates the signal-initiating kinase Lyn, creating receptor binding sites for the signal-propagating kinase Syk. Syk activation leads within minutes to the tyrosine phosphorylation of many proteins, mobilization of cellular Ca^2+^, the release of inflammatory mediators via degranulation and within hours to cytokine production. B) Micrograph of the monolithic microfluidic chip used in this study. The inlets used for individual cell/reagent loading, buffer washing and intracellular immunostaining are indicated in the figure. C) Selected regions for live-cell imaging. Scale bar: 20 µm. D) Hydrodynamic focusing and detection region for μflow cytometry.

We used multivalent dinitrophenyl (DNP)-conjugated bovine serum albumin (DNP-BSA), with each BSA molecule conjugated on average to ∼25 DNP groups, to crosslink DNP-specific IgE bound to cell-surface FcεRI, thereby stimulating FcεRI signaling. Receptor crosslinking initiates a tyrosine kinase cascade and activation of numerous signaling proteins, including phospholipase Cγ (PLCγ) and phosphatidylinositol 3-kinase (PI3K). Important downstream responses include the mobilization of cellular Ca^2+^ as well as the release of inflammatory mediators from preformed stores and *de novo* cytokine production (Figure 1A). As described in the sections that follow, we used the microfluidic device illustrated in Figure 1B to systematically evaluate selected cell signaling events, capturing quantitative measurements on a cell-by-cell basis from a small population of cells (hundreds to thousands). This capability to interrogate signaling at the single-cell level in a small population of cells may be valuable for studies involving rare cell populations, such as those isolated from primary tissues or biopsies, and for studies that rely on expensive reagents.

### Microfluidic device

The microfluidic chip used to monitor the FcεRI pathway is illustrated in Figure 1B. The quartz-based device includes three spiral channels for cell preparation and cellular assays. The spiral geometry was adopted to minimize dead volume [8]. The spiral channels have identical depth (30 µm) but slightly different widths (150–200 µm) and lengths (70–90 mm). Imaging is possible because the glass bottom of the device is polished to 180 µm in thickness. The middle spiral channel, in which we placed cells, has a width of 200 µm and a length of 90 mm. This channel can hold from a few hundred to a few thousand cells depending on the density of the seeding cell suspension. The fluid volume of the middle spiral channel is ∼500 nL.

The device was designed and operated to keep cells viable for long time and to avoid non-physiological shear stresses. When the middle spiral channel was coated with live cells, it was supplied with growth medium at a flow rate of 0.2 µL/min. At this flow rate, a significant fraction of the fluid in the channel (∼40%) is purged and replaced every minute. Shear stresses generated in a microfluidic chip can unintentionally activate cells. In our experiments, the shear stresses experienced by live cells were less than 20 to 40 dyn/cm^2^ for the highest flow rates used (0.5–1.0 µL/min). These shear stresses are similar to those that cells experience in blood vessels (10–40 dyn/cm^2^) [23], [24]. The results of on-chip experiments were validated by conventional assays (e.g., ELISA), and all on-chip experiments were performed at least three times, with relatively low measurement variability from experiment to experiment (see below).

Further notable features of the microfluidic device are as follows. As indicated in Figure 1B, there are inlets that can be used for loading of cells and reagents, buffer washing and immunostaining. The chip is integrated with accessories needed to operate the chip, such as activated pressure controllers, electronic control valves, digital heaters and optical detectors. These components are assembled into a bench-top, automated device that maintains cell viability and enables quantitative measurement of signaling events in single cells using high-resolution imaging and on-chip flow cytometry. The chip is reusable and easy to decontaminate using a standard cleaning protocol (see Materials and Methods). The chip was routinely reused without loss of measurement reproducibility (see below). Our chip design permits end-to-end experimentation, which mitigates manual errors typically associated with conventional assays. The integration of all sample preparation – including cell fixation, membrane permeabilization, immunostaining, on-chip imaging and flow cytometry – into a closed monolithic automated platform increases reproducibility and minimizes labor, contamination, loss of cells and reagents, and variations in cell microenvironment [8], [25], [26]. Individual time courses reported below were generated using a single device, with data collected in real time.

### Measurement of Syk phosphorylation

**Figure 2:**
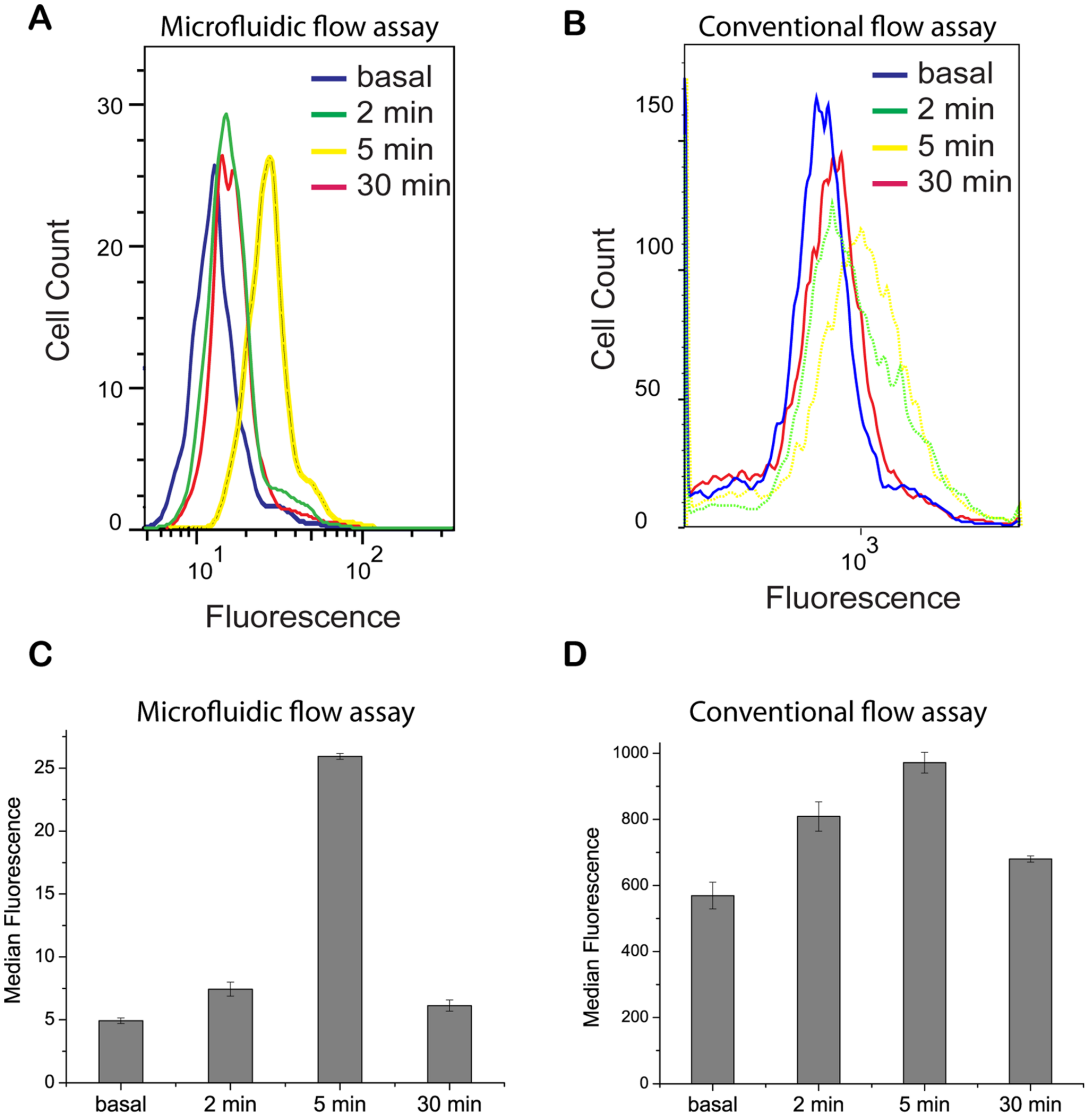
Analysis of phosphorylation of Syk. Phosphorylation at Y346 in Syk (rodent numbering) was assessed at the basal condition and at 2, 5 and 30 min after stimulation with 100 ng/ml of DNP-BSA. Phosphospecific immunostaining was followed by μflow or conventional flow cytometry analysis. A) Histograms from μflow cytometry. B) Histograms from conventional flow cytometry. C) Each bar reports the average and standard deviation of median fluorescence determinations made by μflow cytometry in three independent experiments. D) Same as panel C except median fluorescence determinations were made by conventional flow cytometry.

Protein tyrosine phosphorylation is a prominent feature of FcεRI signaling. The signal-initiating kinase Lyn is activated upon crosslinking of IgE-FcεRI complexes. Lyn mediates phosphorylation of FcεRIγ ITAM motifs, creating dual docking sites for the tandem SH2 domains of Syk, leading to auto-phosphorylation and activation of Syk [27]. Lyn also directly phosphorylates Syk. As mentioned above, we used a multivalent antigen, DNP-BSA, to crosslink cell-surface anti-DNP IgE. We then monitored Syk phosphorylation at the single-cell level for a population of cells as a function of time (Figure 2).

Figure 2A reports the results of using our microfluidic device to measure the kinetics of Syk phosphorylation induced by DNP-BSA treatment. After cells were immobilized on the channel surface and saturated with IgE, we used the device's valve controllers to precisely set the duration of stimulation (2, 5 and 30 min) in separate experiments. Each experiment was replicated three times. After stimulation, cells were fixed, permeabilized and stained with phycoerythrin (PE)-conjugated mouse monoclonal Syk antibody specific for phosphorylated Y346 in Syk (rodent numbering). Finally, the cells were detached (see Materials and Methods) and analyzed via microfluidic flow cytometry (μflow). The efficiency of cell release was greater than 80%. The histograms of Figure 2A show that Syk phosphorylation was detectable within 2 min of stimulation at an antigen concentration of 100 ng/ml. Syk phosphorylation peaked around 5 minutes and then returned toward baseline levels. The distributions have shoulders that are indicative of cell debris. The shoulders capture less than 5% of the area under the curve and are therefore not representative of the cell population assayed.

We note that an alternative to μflow that could have been used to monitor Syk phosphorylation is on-chip single-cell imaging [8], [17]. On-chip single-cell imaging results were not generated for this report for two reasons. First, this imaging capability has already been demonstrated in earlier work [8], [17] and it provides results that are more qualitative than quantitative. Second, and perhaps more importantly, the throughput possible with μflow (hundreds to thousands of cells) is much greater than that possible with on-chip single-cell imaging. This capability is important because we need to probe a large number of individual cells to precisely characterize the heterogeneity of a cell population.

To validate the μflow results (Figure 2A), we also assayed Syk phosphorylation using conventional flow cytometry (Figure 2B). As can be seen, both methods yield the same qualitative results.

To quantify the reproducibility of the μflow results, we determined the median fluorescence intensity for each distribution measured using either μflow or conventional flow cytometry in each of three replicate (independent) experiments at all time points of interest. We then calculated the mean and the standard deviation of the three median values. The results for μflow are shown in Figure 2C and the results for conventional flow cytometry are shown in Figure 2D. As can be seen, the μflow measurements are more reproducible (less variable) than the measurements made using conventional flow cytometry.

To determine if our μflow measurement technique is able to detect changes in Syk phosphorylation across experimental conditions with statistical significance, we asked if measured distributions are distinct by performing Student's *t*-tests on three independent experiments and calculating *p*-values (*N* = 3). We found that the distributions measured at 2, 5, and 30 min are all distinct from the basal distribution (the *p*-values are all less than 0.01). Likewise, the distribution measured at 5 min is distinct from the distributions measured at 2 or 30 min (the *p*-value is less than 0.01 in each case). The distributions measured at 2 and 30 min are not distinct, i.e., the *p*-value for this comparison is greater than 0.01.

A salient point to note about the results of Figure 2 is that while the kinetics of Syk phosphorylation can be measured by Western assays [21], the cell-to-cell variation (Figures 2A and 2B) cannot. Western results can be expected to correlate with the mean of the measurements shown in Figure 2A and would not provide information about sub-populations of cells exhibiting behaviors different from the average.

What is the source of the cell-to-cell variation in Syk phosphorylation seen in Figure 2? In studies of gene regulation, heterogeneity sometimes is seen to arise from fluctuations in small population sizes because of stochastic chemical kinetics [28]. However, this source of heterogeneity, intrinsic noise, may not be relevant in our system because the copy numbers of proteins involved in FcεRI signaling proteins are fairly large, ranging from tens to hundreds of thousands per cell [29]. An alternative possible explanation for the observed heterogeneity is variation in protein expression level from cell to cell [29]. This source of heterogeneity can be viewed as a type of extrinsic noise.

### Modeling of Syk phosphorylation

To investigate the potential roles of intrinsic and extrinsic noise in cell-to-cell variability in FcεRI signaling, we turned to predictive modeling. A model relevant for our experimental setup was formulated by combining two published models, one for DNP-BSA interaction with anti-DNP cell-surface IgE [30] and one for early signaling events, including Syk phosphorylation, triggered by aggregation of FcεRI, which is the first step in FcεRI signaling [29]. In the former model, DNP-BSA is assumed to have two binding sites that alternate between hidden and exposed states and that are each available for interaction with anti-DNP IgE when in the exposed state. This model is consistent with quantitative measurements of DNP-BSA binding to anti-DNP IgE in complex with FcεRI on RBL-2H3 cells [30]. The latter model captures interactions of FcεRI and the protein tyrosine kinases Lyn and Syk. This model is consistent with quantitative Western assays of receptor subunit phosphorylation in RBL-2H3 cells [29].

Our model, which is given in the Supplemental Material in the form of a BioNetGen input file (Text S1), can be simulated using BioNetGen [31]. The parameter values specified in the supplemental file are the nominal values estimated in earlier work. Key parameter values are also summarized in Table 1. Because the earlier modeling work considered RBL-2H3 cells, as we do here, we took the nominal protein copy numbers to be the mean protein copy numbers expected for a population of RBL-2H3 cells. Thus, as in the model of Faeder et al. [29], we assume that the total copy numbers of FcεRI, Lyn and Syk in RBL cells are comparable and large (i.e., on the order of 10^5^ molecules per cell for each protein). However, only a fraction of Lyn (7%) is taken to be available to participate in signaling, based upon evidence that a large fraction of Lyn exists in an autoinhibited conformation [29], [32], [33]. This assumption is a simplification, as the fraction of Lyn available to participate in signaling is regulated and can be expected to change during the response to an antigen stimulus. However, according to a recently developed model for B cell antigen receptor (BCR) signaling, which explicitly accounts for regulation of Lyn autoinhibition [34], the fraction of Lyn in the autoinhibited conformation is expected to remain significant during the response to an antigen stimulus that yields sustained phosphorylation of Syk. Thus, the assumption used here, inherited from the study of Faeder et al. [29], seems adequate. The model of Barua et al. [34], which considers Lyn regulation in the context of BCR signaling, points to how current models for FcεRI signaling could be extended to obtain a more realistic treatment of Lyn regulation in the context of FcεRI signaling in future work.

**Table 1:** Model parameters.(a)

Parameter	Values	Comment
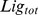	11 nM ( 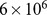 molecules/cell) (b) (c)	DNP-BSA concentration in experiments; a concentration of 11 nM was used in the study of Xu et al. [30].
*Rec_tot_*	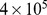 molecules/cell	Average FcεRI concentration [29].
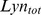	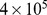 molecules/cell	Average Lyn concentration [29]. Lyn available to participate in signaling is set at 7% of total Lyn.
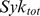	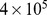 molecules/cell	Average Syk concentration [29].
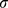	0.2	Each protein copy number is assumed to be distributed log normally around the average concentration. The standard deviation is common for all three distributions by assumption and found through fitting.
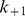	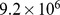 M^−1^s^−1^ ( 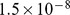 molecules^−1^s^−1^) (c)	Association rate constant for DNP-BSA binding IgE-FcεRI from solution [30].
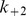	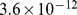 cm^2^s^−1^ ( 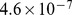 molecules^−1^s^−1^) (d)	Association rate constant for ligand binding IgE-FcεRI on the membrane (receptor crosslinking). In Xu et al. [30] a lumped constant *k_+2_Rec_tot_* = 0.11 was found to be consistent with binding data.
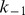	0.012 s^−1^	Dissociation rate constant for DNP-BSA binding IgE-FcεRI from solution [30].
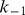	0.012 s^−1^	Dissociation rate constant for DNP-BSA binding IgE-FcεRI on the membrane (receptor crosslinking) [30].
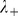	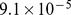 s^−1^	Rate constant for ligand site appearance [30].
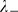	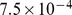 s^−1^	Rate constant for ligand site disappearance [30].

(a) This table only lists values for parameters that characterize DNP-BSA interaction with IgE-FcεRI complexes and cell-to-cell variability in protein expression levels. Other parameter values are the same as those given by Faeder et al. [29].

(b) Values in parentheses are expressed in the unit system recommended by Faeder et al. [57].

(c) Unit conversion is based on a cell density of 10^6^ cells/ml [29].

(d) Unit conversion is based on a cell surface area of
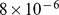
cm^2^ [57].

To assess the potential role of intrinsic noise in FcεRI signaling, we simulated the model deterministically and stochastically and compared the results (Figure S1). Nominal parameter values were used. As expected, because of the large numbers of molecules, stochastic fluctuations are minor and the stochastic and deterministic simulation results are nearly indistinguishable. It seems that the observed cell-to-cell variability in Syk phosphorylation (Figure 2) cannot be explained by intrinsic noise.

The model describes a rapid increase in Syk phosphorylation followed by a plateau in Syk phosphorylation (Figure S1), whereas experimentally we observe an increase at early time-points until a maximum is attained around 5 min followed by a return to a lower a phosphorylation level at 30 min. The model does not describe the decrease at late time-points. This is very probably because the model is formulated to capture the earliest events in FcεRI signaling and does not include many proteins involved in later signaling events (such as receptor internalization, onset of negative regulation) that can lead to reduction in Syk phosphorylation.

**Figure 3:**
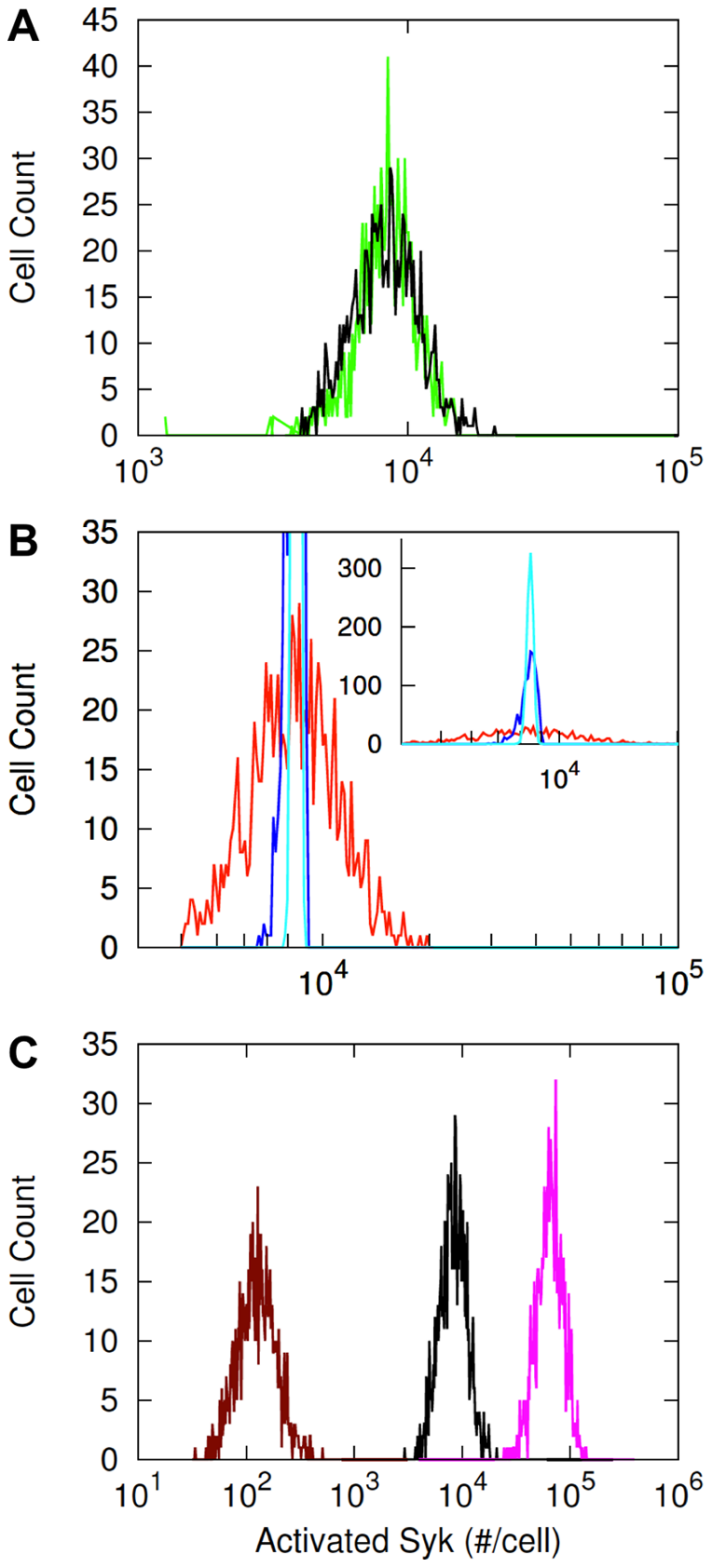
Model-based analysis of Syk phosphorylation. A) Simulated heterogeneity in Syk phosphorylation. The simulated distribution (black line) is comparable to the experimental distribution obtained at either 2 (green line) or 30 min after DNP-BSA stimulation (cf. Figure 2A). The histogram summarizes the predicted steady-state levels of Syk phosphorylation from 10^3^ simulation runs, meaning it represents the distribution of phosphorylated Syk across a population of 10^3^ cells according to the model. In simulations, signaling is induced by a ligand corresponding to DNP-BSA. The copy numbers of the signaling proteins FcεRI, Lyn, and Syk were varied log normally with standard deviation 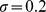 about their nominal means (Table 1). B) The outermost histogram (red line) corresponds to the case where only Lyn copy number is varied, the middle histogram (blue line) corresponds to the case where only receptor copy number is varied, and the innermost histogram (cyan line) corresponds to the case where only Syk copy number is varied. In each case, while the copy number of one protein is varied the copy numbers of the other two signaling proteins are kept constant. C) Predicted effects of Lyn knockdown and Lyn overexpression on the distribution of Syk phosphorylation. The black line represents the wild type case (same as in panel A), the dark red line represents the Lyn knockdown case, and the magenta line represents the Lyn-overexpression case. In the knockdown and overexpression cases, mean copy number of Lyn is taken as 10-fold lower and 10-fold higher than the wild type case. It should be noted that the horizontal axis is logarithmic. Thus, a translation of a given distance from any point along the axis reflects the same fold change but not the same absolute change in copy number.

To assess the potential role of extrinsic noise in FcεRI signaling, we simulated the model repeatedly with copy numbers varying from run to run, with each run simulating the response of a single cell to addition of DNP-BSA. In a given simulation run, the copy numbers used for FcεRI, Lyn, and Syk were each selected from a log-normal distribution centered on the corresponding mean, i.e., each copy number *X* was assigned a value e^μ+σ*Z*^, where μ is the natural logarithm of the mean copy number indicated in Table 1, σ = 0.2, and *Z* is a standard normal random variable. The standard deviation σ, which corresponds to a coefficient of variation (CV) of 0.2, which is similar to that measured in experimental studies of cell-to-cell variability in protein expression levels [29], [35], [36], was determined via a fitting procedure (see Materials and Methods) as the value of σ that best aligns the predicted distribution of Syk phosphorylation (Figure 3A) with the observed distribution of Syk phosphorylation at *t* = 2 (Figure 2B). Additional details about fitting are provided in Materials and Methods. By using the same σ for all protein copy numbers, we are assuming that variations in FcεRI, Lyn and Syk copy numbers arise from a similar process.

To determine which protein copy number has the most influence on variability in Syk phosphorylation, we varied the mean total concentration of each individual protein while keeping the mean concentration of the other two proteins fixed. The results of this sensitivity analysis indicate that variation in FcεRI or Syk level has only a minor effect, whereas variation in Lyn, especially a decrease in Lyn copy number, has a major effect on heterogeneity in Syk phosphorylation (Figure 3B). This result is a consequence of the sum of the nonlinear relationships captured in the model; however, the result is perhaps obtained mainly because in our model only a fraction of total Lyn is taken to be available to participate in FcεRI signaling. In any case, Syk phosphorylation is predicted to be sensitive to changes in Lyn copy number, especially decreases in Lyn copy number. This finding suggested that knockdown of Lyn might lead to an observable difference in the distribution of Syk phosphorylation. Indeed, the model predicts that knockdown of Lyn should increase the width of the distribution of Syk phosphorylation (Figure 3C). It should be noted that this prediction indicates greater fold-changes (vs. absolute changes) in Syk phosphorylation at lowered Lyn copy number.

The prediction of Figure 3C is testable. To test the prediction, one could attempt to leverage natural variability in Lyn copy number and sort cells into subpopulations with relatively low and high Lyn copy numbers based on labeling with a fluorophore-conjugated antibody specific for Lyn. One could also contemplate an approach based on knockdown of Lyn expression through RNA interference. The latter approach has the advantage that a Lyn-specific antibody is not strictly required.

### Measurement of cell degranulation by monitoring Ca^2+^ and β-hexosaminidase release

**Figure 4:**
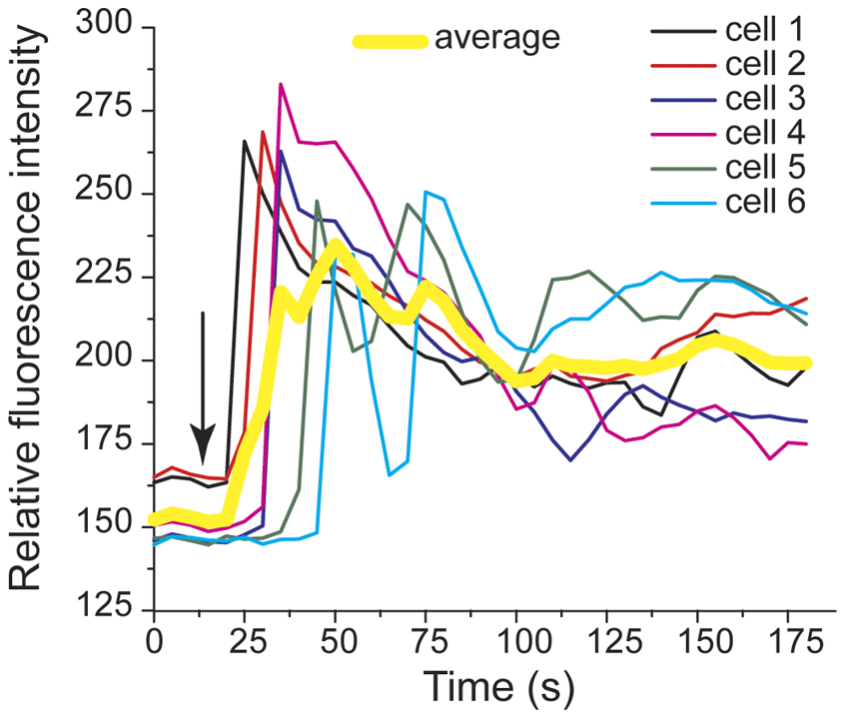
Time course of antigen-stimulated Ca^2+^ mobilization in individual RBL-2H3 cells. Mobilization was assessed in microfluidic channels using calcium indicator Fluo-3. The arrow indicates the time at which the antigen (DNP-BSA) reached the cells in the region of the microfluidic device under observation.

IgE receptor signaling elicits degranulation and release of mediators of inflammation such as β-hexosaminidase and histamine. Prior to degranulation, there is also an increase in the intracellular concentration of Ca^2+^ [37]. We used our device to monitor antigen-induced intracellular Ca^2+^ mobilization and observed considerable cell-to-cell variation as reported previously in the literature [38], [39]. Figure 4A shows time courses for six individual cells and the average of these six time courses. Additional information about single-cell calcium responses is provided in Table 2, which reports the time to onset of calcium mobilization for each of 45 cells.

**Table 2:** The variability of time of onset of the calcium response to antigen (DNP-BSA) stimulation.

Response time (s)	# of responding cells
10	2
13	4
15	6
20	5
23	8
25	6
35	5
45	2
No response	7
Total cells	45

A cell was classified as responding to antigen if a transient increase in fluorescence intensity occurred at least 10% above baseline.

**Figure 5:**
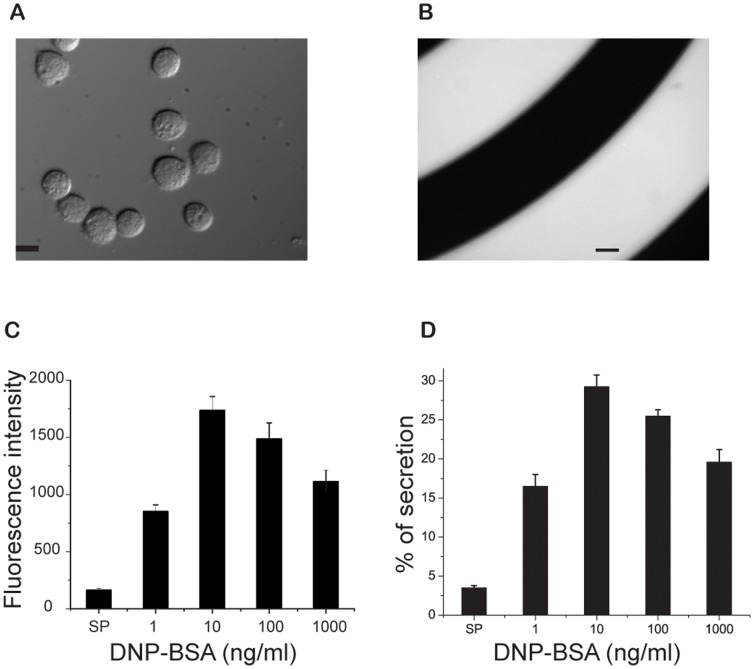
Microscopic measurement of released β-hexosaminidase in microfluidic channels. A) Differential interference contrast (DIC) image of representative live RBL-2H3 cells stimulated with DNP-BSA with no addition of MUG, the fluorogenic substrate of β-hexosaminidase. Scale bar: 10 µm. B) Fluorescence images of live stimulated cells 20 min after addition of both DNP-BSA and MUG. The cells were immersed in supernatant containing released β-hexosaminidase and the fluorescent product methylumbelliferone. The fluorescence signal reveals the spiral geometry of the microfluidic channel. Scale bar: 20 µm. C) A bell-shaped dose-response curve characterizes the degranulation response of live RBL-2H3 cells in microfluidic channels to antigen stimulation. The concentrations of antigen (DNP-BSA) used to stimulate cells ranged from 1 to 1000 ng/ml. D) Results of conventional degranulation assays performed using 24-well tissue culture plates.

We monitored degranulation at the population level using a β-hexosaminidase assay. This assay is based on measuring the activity of released β-hexosaminidase using a fluorogenic substrate, 4-methylumbelliferyl-N-acetyl-β-D-glucosaminide dehydrate (MUG) [40]. Figure 5A depicts a differential interference contrast (DIC) image of RBL-2H3 cells stimulated with 10 ng/ml of DNP-BSA in microfluidic channels. Figure 5B shows fluorescence images of stimulated cells immersed in supernatant containing released β-hexosaminidase and the fluorescent product methylumbelliferone. We found that secretion was >95% complete within 20 min of stimulation. For comparison, Figure 5C reports relative secretion level of β-hexosaminidase over a wide range of antigen doses from 1 ng/ml to 1000 ng/ml of DNP-BSA. β-hexosaminidase activity peaked at 10 ng/ml and decreased as DNP-BSA concentration was increased to 1000 ng/ml, a phenomenon that might be explained by high-dose inhibition of secretion arising from a predominance of monovalent binding [41], [42]. This form of inhibition arises when ligand (antigen) concentration is sufficiently high that receptor sites (i.e., the antigen-combining sites of cell-surface IgE) become saturated, with little ligand-mediated receptor (IgE-FcεRI) crosslinking. In the limiting case of excess ligand, after a transient, most receptor binding sites are occupied by a single ligand molecule, and there is reduced receptor aggregation. As discussed below, an alternative explanation for high dose inhibition may be the active recruitment of inhibitory regulators.

### Measurement of cytokine production

**Figure 6:**
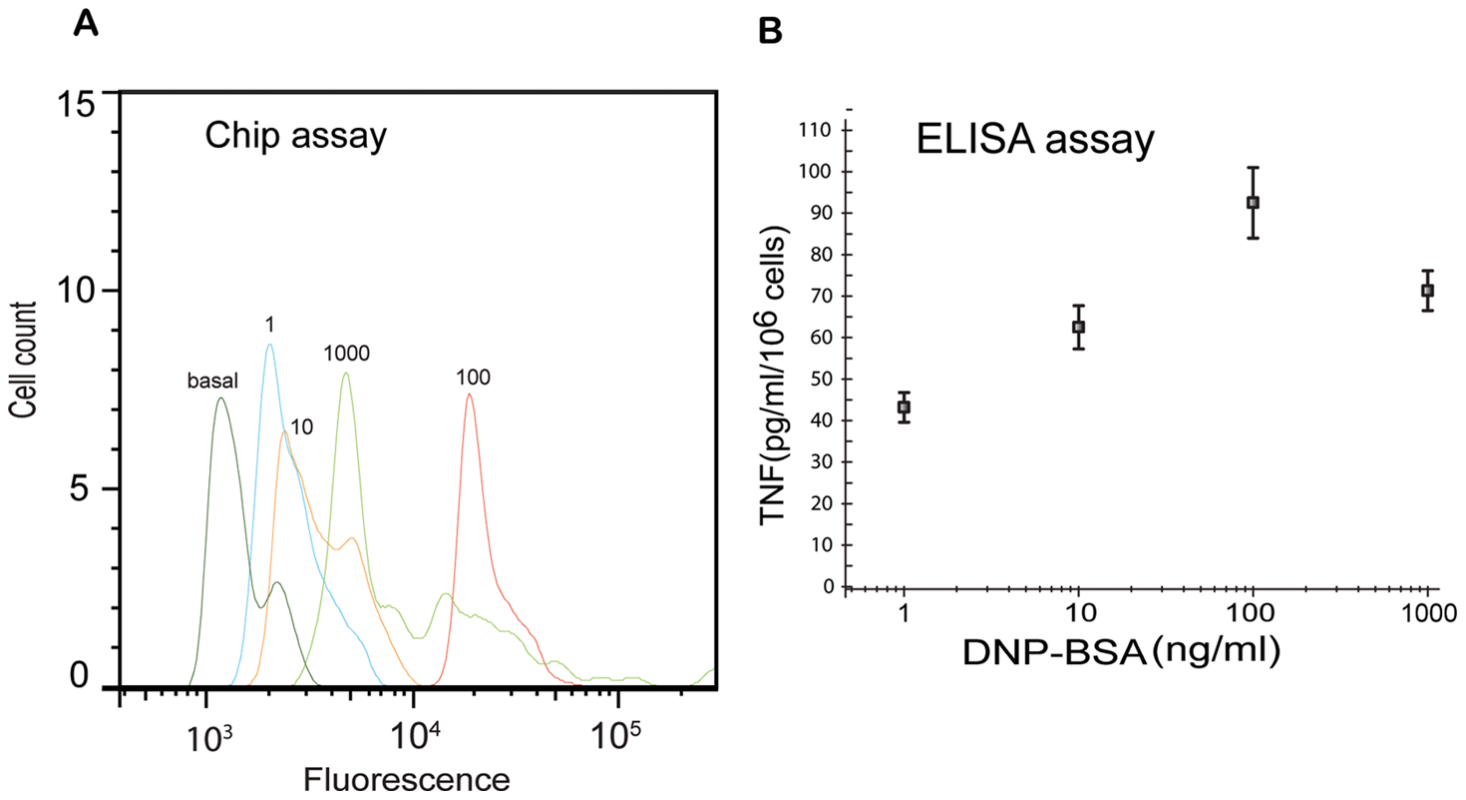
Analysis of TNFα production. Cytokine production by RBL-2H3 cells was measured by μflow cytometry and ELISA after cells were stimulated with doses of DNP-BSA ranging from 1 to 1000 ng/ml. Cells were challenged in the presence of Brefeldin A to block cytokine transport processes. A) μflow cytometric measurements of TNFα production. B) ELISA measurements of TNFα production.

Mast cells and basophils synthesize and release a variety of cytokines, including IL-3, IL-4, IL-5 and TNF, to alter their local microenvironment. Cytokine release leads to the recruitment of inflammatory cells [43], [44]. We used the protein transport inhibitor Brefeldin A to block secretion of cytokines. The resulting accumulation of cytokines inside cells allowed us to assay cytokine levels through immunostaining and μflow cytometry. Measurement of TNFα production revealed the dependence of cytokine synthesis on concentration of DNP-BSA (Figure 6).

Dependence of TNFα production on the dose of DNP-BSA is similar to the dependence of degranulation on the dose of DNP-BSA (cf. Figures 5 and 6). However, the concentration of DNP-BSA that produced maximal degranulation was not equipotent with respect to TNFα production. A 10 fold higher concentration of DNP-BSA was required to elicit maximal cytokine synthesis. ELISA control experiments (Figure 6B) validated the microfluidic results. Microfluidic and ELISA results both indicate that maximum TNFα production (92.5±8.5 picogram per 10^6^ of RBL-2H3 cells) occurs at a DNP-BSA dose of 100 ng/ml.

Several factors could contribute to this phenomenon. First, it is known that pathways bifurcating from FcεRI mediate both positive and negative signaling [45], [46], [47]. Although the classical example of Lyn-mediated negative signaling occurs through the mechanism of phosphorylation of immunoreceptor tyrosine-based inhibitory motifs (ITIMs) in co-receptors [35], [48], new evidence supports the concept that Lyn also mediates negative signaling through incomplete phosphorylation of ITAMs [49]. An important outcome of incomplete ITAM phosphorylation is the recruitment to the membrane of inhibitory signaling molecules, such as SHIP (SH2-containing inositol phosphatase). Further studies are needed to evaluate the link between antigen dose and the bias towards positive or negative signaling. A second factor that may explain the observed requirement for a relatively high ligand dose to achieve maximal cytokine production is dose-dependent activation of Fyn (vs. Lyn), a Src-family kinase that has been implicated in control of cytokine production [45], [50], [51].

## Conclusion

The IgE receptor (FcεRI) signaling pathway plays an important role in allergic diseases. We used an integrated microfluidic device to interrogate the FcεRI signaling pathway with single-cell resolution while minimizing experimental variations. The purpose of our study was twofold. First, we intended to demonstrate capabilities to measure and model IgE receptor signaling at the single-cell level. Second, we intended to characterize cell-to-cell variability in IgE receptor signaling. Heterogeneity in individual cellular responses to stimulation of FcεRI signaling was apparent from the measurements made possible using our microfluidic device. A model-based analysis of the observed heterogeneity in Syk phosphorylation indicated that the heterogeneity could be explained by cell-to-cell variation in total protein copy numbers (i.e., extrinsic noise) but not by intrinsic noise (i.e., stochastic fluctuations arising from small population sizes). Moreover, the observed heterogeneity is predicted to depend more sensitively on cell-to-cell variability in Lyn copy number than on cell-to-cell variability in other protein copy numbers. This prediction is consistent with the concept in the literature that Lyn is limiting. Confirmation of this prediction would suggest that not all possible points of (drug) intervention in a signaling pathway are equally sensitive and moreover that the most sensitive points for intervention could potentially be revealed by measurements of cell-to-cell variability. The variability that would perhaps be most important to measure is variability in signaling readouts (e.g., Syk phosphorylation) rather than variability in total protein copy numbers, because in our model, variability in total Lyn copy number is the same as variability in total Syk copy number. We further found that the multivalent antigen used to crosslink FcεRI was not equipotent between stimulating maximal degranulation and maximal cytokine production. Our study demonstrates the feasibility of using microfluidic technology to quantitatively characterize FcεRI signaling events at the single-cell level to capture the heterogeneity within a population of cells. In future work, our device could potentially be used to study low-abundance cells such as human basophils in blood or rare cells in tissue samples.

## Materials and Methods

### Reagents and materials

Mouse monoclonal anti-DNP-IgE was prepared as described by Liu et al. [52]. DNP-BSA (bovine serum albumin coupled to an average of 25 2,4-dinitrophenyl (DNP) groups per BSA) and Fluo3-AM were purchased from Invitrogen (Carlsbad, CA). Staining buffer, Golgiplug inhibitor, fixation and permeabilization kit and hamster anti-rat tumor necrosis factors (TNF), phycoerythrin (PE)-conjugated anti-rat TNF and recombinant TNF and PE-conjugated mouse monoclonal Ab specific for Y319 in human ZAP-70 and Y352 (Y346) in human (rat) Syk, a substrate of Src family kinases in RBL-2H3 cells [53], were purchased from BD Biosciences (San Jose, CA). Phosphorylation of Y346 in Syk has a positive effect on FcεRI-mediated secretory responses in RBL-2H3 cells [54]. Methylumbelliferyl-N-acetyl-d-glucosaminide dihydrate (MUG) was purchased from Sigma (St. Louis, MO). Active pressure controllers (APC) were purchased from Parker Precision Fluidics (Cleveland, OH).

### The integrated microfluidic platform

Monolithic microfluidic chips were custom fabricated by Caliper Life Sciences (Mountain View, CA) as described in detail elsewhere [8], [55]. A delrin polymer manifold with O-ring seals was used to provide an interface between a chip and fluid reservoirs. Several active pressure controllers were assembled to pressurize the sample reservoir using either N_2_ or air (5% CO_2_) to deliver regents into the channels through PEEK tubings (125 µm i.d.). Air (5% CO_2_) was used when the device was loaded with live cells, which helped to regulate the pH and maintain cell viability. Incubation temperature was monitored using a thermistor attached to the chip manifold. All fluid reservoirs were pressurized by individual APC controllers to ensure precise control of hydrodynamic focusing and cell movement. An in-house engineered rabbit board provides the power for the APC as well as the control voltages via D/A converters. The feedback voltage is sensed using A/D converters. A graphical user interface (GUI) was programmed to interface the rabbit control box with pressure control units and electronic valves.

### Cell activation in microfluidic channels

RBL-2H3 cells were cultured as previously described [56]. Prior to on-chip assays, the microfluidic chips were cleaned using the following (standard) protocol: chips were flushed with 10% freshly prepared bleach solution for 5 min, sterilized with 70% ethanol for 10 min, and then rinsed thoroughly with Hank's buffered saline solution (HBSS) for an additional 10 min. To enhance the adherence of RBL-2H3 cells to the glass surface, 10 µg/ml of human plasma fibronectin in HBSS was delivered into the channel [26], followed by physical adsorption under static conditions for 1 h at room temperature. RBL-2H3 cells in suspension were injected into the device to achieve ∼1000 cells in the middle spiral channel. After a 5 min static incubation of cells at 37°C, cell growth medium was constantly exchanged at a flow rate of 0.2 µL/min for 30 min. Antigen-specific IgE (100 ng/ml monoclonal anti-DNP) was perfused to the adhered cells for 15 min, with an additional 15 min of static incubation to saturate FcεRI receptors on RBL-2H3 cells. Excess IgE was removed by flowing HBSS for 15 min. Flow rates between 0.5 and 1.0 µL/min were used in the washing steps throughout this report, unless mentioned otherwise. Once in place for assays, cells could be kept viable at 37°C for 12 hours with continuous exchange of medium.

### Phosphorylation assay

IgE-FcεRI complexes were crosslinked with 100 ng/ml of DNP-BSA. At different RBL-2H3 activation times, cells were fixed and permeabilized with PBS containing 2% paraformaldehyde (PFA) and 0.5% Tween 20 for 20 min at room temperature. Cells were then stained with PE conjugated phospho-ZAP-70/Syk antibody (1∶100) in Pharmingen staining buffer for 30 min, followed by continuous washing for 15 min with the same buffer. Stained cells were detached from the channel surface after immersion in 1% trypsin-EDTA (vs. 0.025% typically used in cell subculture) at 37°C for 5 min using a high-pressure flow (4 to 5 psi). Detached cells were then hydrodynamically focused into a single file and sent downstream to the detection point of a μflow cytometer. A 488 nm solid state laser was used to interrogate individual cells. Forward scattering was detected through an optical fiber (JTFSH 600 µm core, Polymicro Technologies, Phoenix, AZ) and a photomultiplier (H5784-20; Hamamatsu, Bridgewater, NJ). Emission was passed through a bandpass filter and detected with a second photomultiplier. The output signals were digitized with a USB data acquisition module (NI USB-6009, National Instruments) using a LabVIEW control program (National Instruments). Peak information was extracted and converted into a binary file format FCS for FlowJo (Tree Star, Ashland, OR) analysis. A BD flow cytometer was used to validate our microfluidic measurements using methods described earlier [8].

### Model formulation

As indicated below, we modified a published model for early events in FcεRI signaling, namely, the model of Faeder et al. [29]. This model tracks the interactions of three signaling proteins: FcεRI; Lyn, a membrane-associated Src-family protein tyrosine kinase; and Syk, a cytosolic protein tyrosine kinase. The model encompasses activation of Syk, which is required for secretory responses mediated by aggregation of FcεRI. This model was formulated using the rule-based modeling approach [57], wherein graphs are used to represent proteins and their component parts (e.g., domains) and graph-rewriting rules are used to represent molecular interactions and their effects (e.g., phosphorylation).

According to the model, FcεRI signaling is initiated upon aggregation of FcεRI. Receptor aggregation enables Lyn, which is weakly and constitutively associated with FcεRI, to trans-phosphorylate the immunoreceptor tyrosine-based activation motif (ITAM) tyrosines of the β and γ subunits of FcεRI. The phosphorylated β ITAM interacts with the SH2 domain of Lyn, enhancing association of Lyn with the receptor. The phosphorylated γ ITAM tyrosines interact with the tandem SH2 domains of Syk, allowing recruitment of Syk to the membrane and phosphorylation of Syk by Lyn and by co-localized copies of itself. Thus, the model accounts for two types of tyrosines in Syk: those phosphorylated by Lyn and those phosphorylated by Syk. Because phosphorylation of Y346 in Syk, (rodent numbering), which is measured in experiments using an antibody specific for pY346, is ablated by an inhibitor of Src-family kinases but not by an inhibitor of Syk [58], we consider only model-predicted Lyn-mediated phosphorylation of Syk when comparing predicted and measured Syk phosphorylation levels. We modified the original model, in which soluble chemically-crosslinked dimers of IgE mediate FcεRI dimerization, by replacing the original rules for ligand-receptor interactions with new rules. These new rules represent interactions of IgE-FcεRI complexes with DNP-BSA in accordance with the model of Xu et al. [30].

In the model of Xu et al.[30], DNP-BSA is taken to have only two effective binding sites when interacting with anti-DNP IgE coupled to FcεRI on the surface of RBL-2H3 cells [30]. According to the model, the two binding sites of DNP-BSA are either hidden or exposed. Only a site in the exposed state is available for interaction with cell-surface IgE.

In our model, we consider heterogeneity in protein expression levels as follows. Faeder et al. [29] assumed a total copy number of 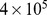 molecules per cell for FcεRI, Lyn, and Syk, but with only 7% of Lyn available to participate in signaling (Table 1). We take 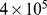 molecules per cell to be the mean total copy number for each signaling protein across cells considered in our study, but we allow copy number to vary log-normally around the mean with a common standard deviation, which is determined through fitting (see below). It should be noted that the copy number distribution for the fraction of Lyn available to participate in signaling has the same shape as that for total Lyn, which is a property of the log-normal distribution [59]. The standard deviation was adjusted, along with a scaling parameter that was used to relate measured fluorescence to predicted protein copy number, to obtain a predicted distribution of Syk phosphorylation as close as possible to the measured distribution of Syk phosphorylation at time *t* = 2 min Additional details about the fitting procedure are given below.

### Model simulation

The model was formulated as a set of rules, encoded using the BioNetGen language [31], [57], for the interactions of DNP-BSA, IgE-FcεRI, Lyn and Syk. The model is provided as a BioNetGen input file, a plain-text file, in the supplementary material (Text S1). The model was simulated deterministically via the generate-first approach [29], [31], meaning that the ordinary differential equations (ODEs) implied by the rules of the model were first found and then these ODEs were integrated numerically using an ODE solver in the CVODE package [60], which is a component of BioNetGen [57]. Likewise, the model was simulated stochastically using an efficient variation of Gillespie's method [61]. We used BioNetGen for network generation and both deterministic and stochastic simulations; default settings were used for algorithmic parameters [57]. The network generated by BioNetGen encompasses 380 chemical species, corresponding to 380 ODEs, and 3862 unidirectional reactions, corresponding to 3862 terms for reaction rates on the right-hand side of the ODEs. This network is larger than the network considered earlier for receptor aggregation induced by IgE dimers [29], which encompasses 354 species, because the version of the model considered here takes into account a different ligand [30].

### Fitting

We compared measured and predicted distributions of Syk phosphorylation by taking measured fluorescence to be proportional to predicted phosphorylated protein copy number. In Figure 3A, we compare 1,000 fluorescence measurements (reflecting Syk phosphorylation levels in individual cells) and predicted Syk phosphorylation levels for 1,000 virtual cells, which are reported in units of number of phosphorylated Syk molecules per cell. We converted each measured fluorescence level (a.u.) to a copy number (number of molecules per cell) by multiplying fluorescence level by a constant scaling parameter. The value of this parameter (1,258 molecules per cell per a.u.) was selected so that the measured and predicted distributions of Figure 3A match as closely as possible. In evaluating the match, we used data binning, i.e., we compared actual and virtual cell counts at discretized Syk phosphorylation levels. Each continuous-valued Syk phosphorylation level, obtained either from our model or from scaling of fluorescence measurements, was mapped to the nearest of 300 bins. On a logarithmic scale, the 300 bins evenly and finely divided the interval from minimum to maximum predicted Syk phosphorylation level. In the fitting procedure, two parameter values were varied: the scaling parameter that relates fluorescence to protein copy number and σ, the standard deviation commonly used in all log-normal distributions for protein copy numbers. Parameter values were varied and quality of fit was assessed through a brute-force approach (i.e., grid search).

### Calcium mobilization assay

Calcium indicator Fluo-3 acetoxymethyl ester (AM) was dissolved in dimethyl sulfoxide (DMSO) and mixed with 20% pluronic acid. This stock solution was then diluted with HBSS to achieve final concentrations of Fluo-3 and pluronic of 4 µM and 0.02% (w/v), respectively. Fluo-3 was loaded into the channel that was seeded with cells for 10 min at 1 µL/min, with an additional 15 min of static incubation in the dark. Excess fluo-3 was removed by 15 min of continuous washing. Time lapse fluorescence images were acquired with an ORCA R2 CCD camera (Hamamastu, Bridgewater, NJ) once 100 ng/ml of DNP-BSA was injected into a chamber at 2.5 µL/min. The camera was interfaced with a Metamorph imaging system (Molecular Devices) on an IX-71 Olympus microscope with an exposure time of 100 ms. Data were analyzed using the Metamorph software.

### Degranulation assay

Degranulation assays were performed in a microfluidic channel to monitor the release of the chemical mediator β-hexosaminidase. A series of mixtures of DNP-BSA with varying concentrations of DNP-BSA (1–1000 ng/ml) and MUG (1 mM) in HBSS was gradually delivered into the channel for 5 min, followed by an additional 15 min of static incubation at 37°C. Fluorescence images of the hydrolyzed substrates were measured with 360 nm excitation and 450 nm emission filters (Semrock, Rochester, NY). To avoid fluorescence saturation, an optimal image acquisition protocol was determined as 50 ms exposure time using a 10× objective. The intensity of individual samples was analyzed using the Metamorph software. Background fluorescence with substrate in HBSS buffer was subtracted from all images. To validate the microfluidic results, degranulation assays were carried out on 24-well culture plates using a standard protocol [56].

### Cytokine assay

DNP-BSA activated RBL-2H3 cells were stained and analyzed with our μflow cytometer using the aforementioned kinase assay protocol with the following two modifications: 1) DNP-BSA was used to challenge cells for 2 hr at 37°C in the presence of Golgiplug containing Brefeldin A, a protein transport inhibitor that blocks cytokine transport process, and 2) cells were stained with PE-conjugated hamster anti-rat TNFα at room temperature for 30 min. The specificity of staining was verified by pre-blocking anti-TNF antibody with recombinant TNF and by pre-incubation of cells with unlabeled TNF antibody as described in the BD Pharmingen manufacturer's protocol. To validate the microfluidic results, TNF in supernatant was quantified using the BD OptEIA rat TNF ELISA set according to the manufacturer's recommended protocol.

## Supporting Information

Figure S1 **Comparison of stochastic and deterministic simulations results for Syk phosphorylation.** The black and red lines represent time-dependent phosphorylation of Syk obtained by simulating the model deterministically and stochastically, respectively. Simulations are based on nominal parameters (Table 1); total protein copy numbers are set at their nominal mean values (Table 1). The phosphorylation curves in both cases correspond to stimulation with a ligand concentration of 10 nM.
(TIFF)

Text S1 **BioNetGen model input file.**
(BNGL)
