# Supplementary figures and images for: Single-Cell Measurements of IgE-Mediated FcεRI Signaling Using an Integrated Microfluidic Platform
Source: PLoS One. 2013 Mar 27;8(3):e60159. doi: 10.1371/journal.pone.0060159 (PMC3609784; doi:10.1371/journal.pone.0060159)

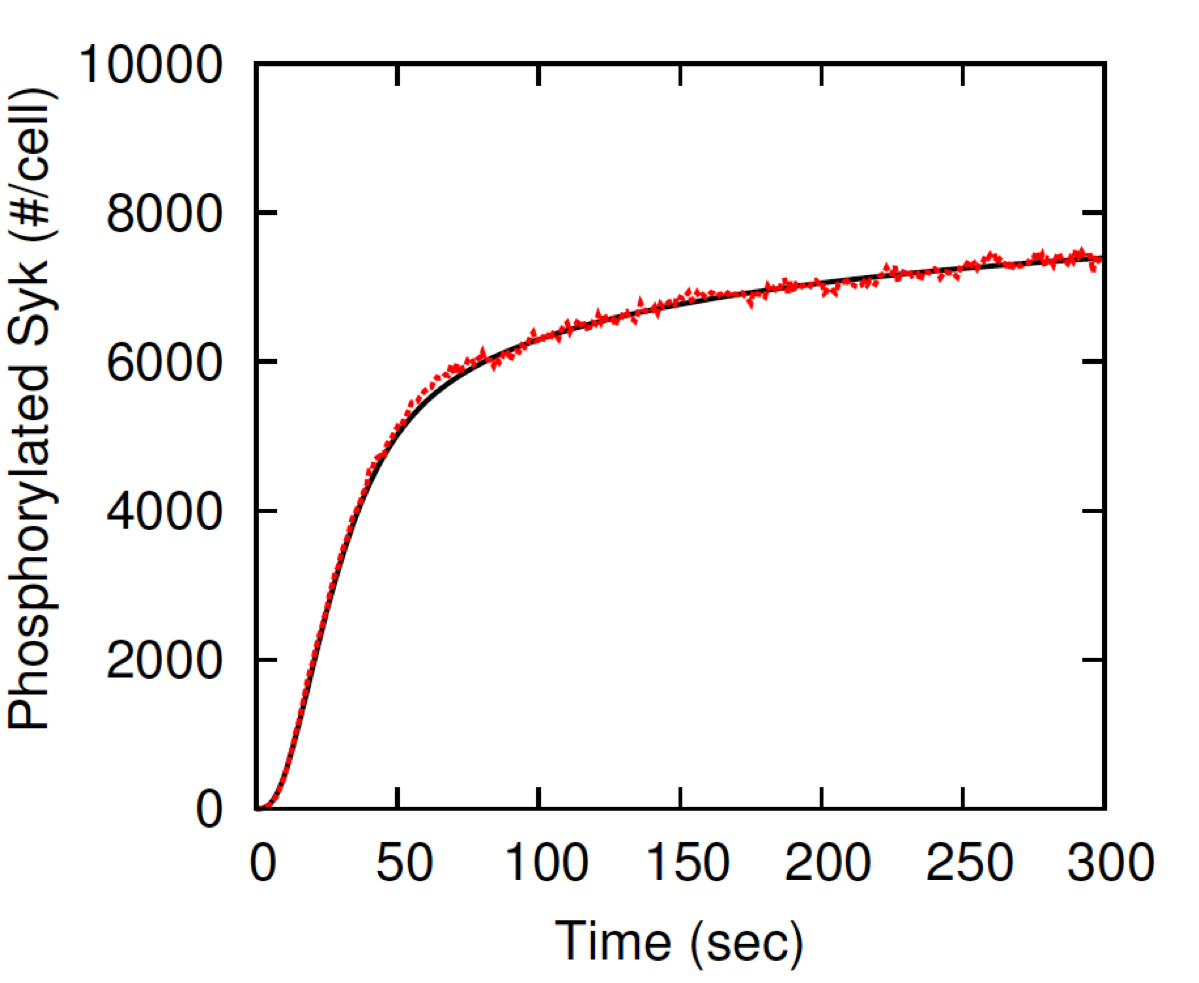

Supplement: Figure S1 — Comparison of stochastic and deterministic simulations results for Syk phosphorylation. The black and red lines represent time-dependent phosphorylation of Syk obtained by simulating the model deterministically and stochastically, respectively. Simulations are based on nominal parameters (Table 1); total protein copy numbers are set at their nominal mean values (Table 1). The phosphorylation curves in both cases correspond to stimulation with a ligand concentration of 10 nM. (TIFF) [file pone.0060159.s001.tiff]
